# Medication-Related Osteonecrosis of the Jaw with Spontaneous Hemimaxilla Exfoliation: Report of a Case in Metastatic Renal Cancer Patient under Multidrug Therapy

**DOI:** 10.1155/2020/8093293

**Published:** 2020-10-22

**Authors:** F. Bennardo, C. Buffone, D. Muraca, A. Antonelli, A. Giudice

**Affiliations:** School of Dentistry, Department of Health Sciences, Magna Graecia University of Catanzaro, Viale Europa, Catanzaro 88100, Italy

## Abstract

Medication-related osteonecrosis of the jaw (MRONJ) is a well-recognized complication of drug therapies for bone metabolic disorders or cancer related to administration of antiresorptive (bisphosphonates and denosumab) and antiangiogenic drugs. This report describes an advanced and unusual case of stage III peri-implantitis-induced MRONJ involving the right upper jaw which was attempting to self-exfoliate. A 61-year-old male patient, rehabilitated with the placement of two implants when he was still healthy, was suffering from metastatic renal cancer previously treated with bevacizumab, interleukin-2, zoledronic acid, denosumab, cabozantinib and nivolumab. He had been under treatment of nonsurgical therapy over a year, based on antibiotic and antiseptic mouth rinse, without improvement of oral conditions. Surgical treatment consisted of massive sequestrectomy and complete surgical debridement of necrotic bone tissues. The specimen was sent for histopathologic analysis, which confirmed bone tissue necrosis with no evidence of metastatic disease. Two-month follow-up revealed a considerable life quality improvement. Although this complication is well known, the uniqueness of this case is given by its severity, related to the administration of multiple antiresorptive and antiangiogenic drugs, by the natural response of the oral cavity with the almost complete self-exfoliation of the massive necrotic zone. This case is emblematic in highlighting the controversies in the management of MRONJ, which certainly require effective collaboration of the multidisciplinary health care team that could improve patient safety and reduce the risk of developing MRONJ.

## 1. Introduction

Medication-related osteonecrosis of the jaw (MRONJ) is a well-recognized complication of drug therapies for bone metabolic disorders or cancer, defined as a persistent bone exposure within the oral cavity for a minimum period of 8 weeks in patients without a history of radiotherapy in the head and neck region. Antiresorptive drugs, such as bisphosphonates (BPs) and denosumab, are successfully used in low-dose therapy to prevent bone pathological fractures in patients with osteoporosis and to treat other bone metabolic diseases such as Paget's disease and in high-dose therapy to prevent bone metastasis in patients with cancer [1].

A recent literature review identified a wide range of medications classified as tyrosine kinase inhibitors, monoclonal antibodies, mammalian target of rapamycin inhibitors, radiopharmaceuticals, selective estrogen receptor modulators, and immunosuppressants that have been implicated in MRONJ in addition to the drugs already mentioned [2].

MRONJ treatment initially involved nonsurgical therapy in mild cases and surgical therapy in severe cases, but currently, the indication for surgical therapy seems to prevail even in less severe cases [3, 4].

This article presents an unusual and extreme case of MRONJ triggered by peri-implantitis in a metastatic renal cancer patient under multidrug therapy.

## 2. Presentation of Case

A 61-year-old male was referred in October 2019 to the Academic Hospital of Magna Graecia University of Catanzaro by his family dentist for the evaluation and treatment of a probable upper jaw osteonecrosis.

His medical history revealed he was under treatment since April 2017 for a metastatic renal carcinoma with brain and vertebral osseous metastasis without jaw involvement. Additional medical conditions included arterial hypertension (treated with enalapril 10 mg twice daily), hypothyroidism (treated with levothyroxine 60 mg daily), and no history of smoking.

Starting from May 2017, he received interleukin-2 (500.000 UI) subcutaneously 3 weeks a month, twice a day for 5 days a week. From June 2017, bevacizumab (25 mg/ml), every 2 weeks, for 6 months, was administered. He had also undergone a monthly 4 mg infusion of zoledronic acid (4 mg) for 5 cycles which was then replaced, in December 2017, by denosumab (120 mg) every 4 weeks, until September 2019. Moreover, from May to September 2019, he also received nivolumab (10 mg/ml). Now, he is in treatment only with daily oral doses of cabozantinib (60 mg).

He had been on treatment of nonsurgical therapy over a year (amoxi/clav 875 mg/125 mg, 2 times a day; metronidazole 500 mg, 3 times a day; and antiseptic mouth rinse with chlorhexidine 0.12%, 2 times a day) without improvement of oral conditions. Periodic follow-up had not been performed by his family dentist.

**Figure 1:**
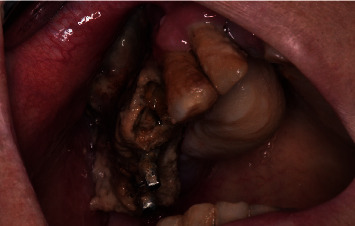
Patient's preoperative clinical condition: necrotic bone exposure and purulent exudate in quadrant I.

At clinical examination, two implants placed by his general dentist in the upper right jaw were found, only with the abutments of a cemented prosthesis (this is no longer present in the oral cavity). The implant rehabilitation, not combined with normal teeth, had been performed, in June 2015, before developing renal carcinoma and starting medical therapy, with two-stage surgical approaches. A partially edentulous maxilla with a totally exposed and necrotic sequestrum in the right hemimaxilla was observed, and there were signs of active infection and purulent exudate with no evidence of fistula formation. Spontaneous avulsion of a tooth was detected laterally to the right hemimaxilla. No implants had been lost prior to decementation of the prosthesis (Figure 1).

Objective exam also revealed a moderate tissue swelling and an asymmetrical face aspect. The patient reported apparently spontaneous painful symptoms. Exposed necrotic tissue emanated a persistent fetor. The patient did not remember when the osseous tissue exposure had begun because in the initial phase, the exposed bone did not give any discomfort. There were no signs of other pathologies. Signs of peri-implantitis with clinical inflammation and peri-implant bone loss were detected.

Various radiological tests have been performed, and an X-ray of the brain has revealed the presence of various brain metastatic lesions.

**Figure 2:**
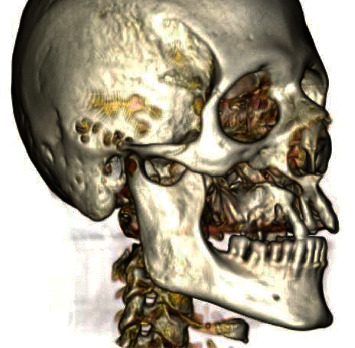
Three-dimensional reconstruction of CT scans that highlighted the detachment of the right hemimaxilla.

Computerized axial tomography with three-dimensional reconstruction revealed an extensive structural destruction of the right maxillary bone (Figure 2) with fractures and continuous solution of the anterior, inferior, lateral, and medial walls of the maxillary sinus. The lesion also involved the hard palate, with bone detachment of the right maxillary arch. Diagnosis of stage III MRONJ according to American Association of Oral and Maxillofacial Surgeons (AAOMS) classification was done.

Considering the severity of the clinical conditions, the patient received prophylactic antibiotic therapy with amoxi/clav 875 mg/125 mg. On the same day, the patient underwent surgery under local anesthesia.

Bone sequestrectomy was performed involving the entire right hemimaxilla held in place by inflammatory tissues. Removal of the sequestrum exposed the ipsilateral nasal cavity. Therefore, careful curettage was performed in order to remove granulation tissues and fragments of the residual necrotic bone.

**Figure 3:**
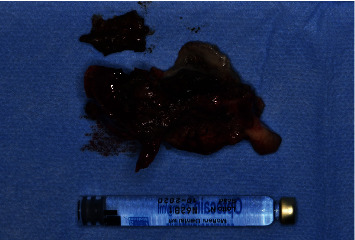
The wide bone sequestration of the right hemimaxilla obtained after sequestrectomy.

The obtained surgical sample (Figure 3) was sent for histopathologic analysis, which revealed “compact bone tissue with morphological aspects coherent with the proposed diagnosis of osteonecrosis.”

Medical therapy with antiseptic mouth rinse (nonalcoholic chlorhexidine 0.12% at least 2 times a day) and systemic antibiotic administration with amoxi/clav 875 mg/125 mg (2 times a day) and metronidazole 500 mg (3 times a day) for 2 weeks after surgery was suggested. The patient was discharged with strict advice to maintain a liquid diet for 2 weeks and an accurate oral hygiene.

**Figure 4:**
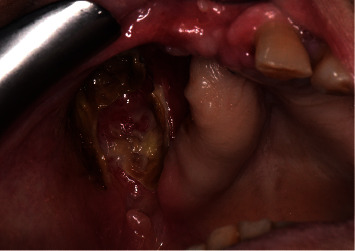
Patient's clinical condition at 2-month follow-up visit: persistence of oroantral communication and presence of mucus.

After 2 months of follow-up, persistence of oronasal antral communication and presence of mucus (discharged from nasal cavity) in the postoperative site were observed (Figure 4). No further regions of bone necrosis were found, and the patient was sent to his dentist for rehabilitation with an obturator prosthesis.

Unfortunately, the prosthetic rehabilitation was never finalized because the patient's condition deteriorated, and he died.

## 3. Discussion

MRONJ lesions induced by pamidronate and zoledronate were first reported by Marx in 2003 in oncologic patients [5]. The risk of developing MRONJ and the response to treatment are mainly influenced by the type and dose of drug therapy, in relation to the patient's primary disease [1]. With the recent development of antiresorptive medications and antiangiogenics used alone or in combination, it seems to be important to understand the molecular mechanisms that lead to MRONJ: the evidence-based mechanisms of pathogenesis include disturbed bone remodeling, inflammation or infection, altered immunity, soft tissue toxicity, and angiogenesis inhibition [6].

The guidelines for MRONJ treatment, assessed by the AAOMS in 2014, suggested a conservative nonsurgical treatment for early stages (I and II) of MRONJ and surgery for advanced stages (III) and mild stages refractory to the nonsurgical approach [1].

Although nonsurgical conservative therapy could be successful to control pain and reduce infection, the high success rate of tissue healing was reported in literature after surgical necrotic bone resection and also in mild stage, improving clinical condition of the patient often expecting downstaging of the lesions [3, 4, 7–12].

In many cases, surgical treatment of stage III MRONJ patients leads to oroantral communication in the posterior maxilla. The closure of these defects represents an additional challenge to the oral surgeon and is essential to improve the long‐term life quality [13].

In recent years, pedicled buccal fat pad (PBFP), combined with ultrasonic bone surgery and L-PRF, has shown some effectiveness in exposed bone coverage and soft tissue healing at the posterior maxillary region. This technique supplies a rich vascular source of adipose-derived adult stem cells that could contribute to the esthetic healing of mucosal defects, prevent further bone weakening, and maximize the success for prosthetic rehabilitation [14].

The treatment of patients with a medical history of malignant diseases and a high-dose drug administration is more complicated with a slower tendency to full healing [15].

To date, many different surgical approaches have been proposed in the treatment of MRONJ lesions: laser therapy, piezoelectric surgery, use of fluorescence for the identification of healthy bone margins, and application of platelet concentrates have become important tools to minimize the invasiveness of surgical therapy and improve tissue healing [15–18].

The patient described in this case report had a metastatic renal cancer treated with multidrug therapy. He was initially managed with nonsurgical therapy that could have eased the halitosis and pain, for more than one year, without an improvement of clinical conditions and with a worsening of MRONJ stage and quality of life. Association of BPs and antiangiogenic drugs leads to more severe and frequent cases of MRONJ than BPs alone [19]. Furthermore, the replacement of a zoledronic acid with denosumab is an additional risk factor for the development of MRONJ [20].

When the patient came to our attention, the surgical therapy choice was mandatory with the removal of the wide sequestrum.

This case is emblematic in pointing out controversies in MRONJ management according to Schiodt et al [4]:

- Early surgical intervention on localized disease may prevent progression and the need for subsequent extensive surgery
- Accurate risk assessment with evaluation of antiresorptive and antiangiogenetic therapies is mandatory before starting MRONJ lesion treatment

In our opinion, the lack of interdisciplinary collaboration and accurate follow-up played a key role in the development of this advanced stage of MRONJ case. The role of general dental practitioners, maxillofacial and oral surgeons, and oncologists in this area must be regularly checked for oral and dental health and must be motivated before starting antiresorptive and antiangiogenic drugs.

Patients continue to be at risk of developing MRONJ with a significant detrimental impact on quality of life due to limited preventive multidisciplinary interventions. MRONJ-education programs and an effective collaboration with the other professional groups could potentially reduce the risk of MRONJ and improve patient safety [21].

## 4. Conclusion

In conclusion, though surgical therapy already in the early stages of MRONJ might prevent the progression of the disease, a multidisciplinary approach to the prevention of MRONJ is essential to optimize high-risk cancer patient management and to improve quality of life.

This outcome, in addition to a better communication among dentists and other health care personnel experienced is the best choice both for primary prevention (improvement of periodontal and peri-implant status and restoration of compromised teeth) and for secondary prevention with follow-up in order to identify MRONJ lesions in the early stage.
